# Effects of community-based rehabilitation on caregivers of people with schizophrenia in Ethiopia in the RISE trial

**DOI:** 10.1186/s12888-025-06651-4

**Published:** 2025-03-11

**Authors:** Lotte G. Dijkstra, Helen A. Weiss, Rahel Birhane, Girmay Medhin, Mary de Silva, Charlotte Hanlon, Abebaw Fekadu, Laura Asher

**Affiliations:** 1 London School of Hygiene and Tropical Medicine, London, UK; 2 MRC International Statistics and Epidemiology Group, London School of Hygiene and Tropical Medicine, London, UK; 3 Department of Psychiatry, School of Medicine, College of Health Sciences, WHO Collaborating Centre for Mental Health Research & Capacity Building, Addis Ababa University, Addis Ababa, Ethiopia; 4 Akililu Lemma Institute of Pathobiology, Addis Ababa University, Addis Ababa, Ethiopia; 5 Department of Health and Social Care, London, UK; 6 Centre for Innovative Drug Development and Therapeutic Trials for Africa, Addis Ababa University, Addis Ababa, Ethiopia; 7 Centre for Global Mental Health, Department of Health Service and Population Research, Institute of Psychiatry, Psychology and Neuroscience, King’s College London, London, UK; 8 Department of Global Health & Infection, Brighton and Sussex Medical School, Brighton, UK; 9 Nottingham Centre for Public Health and Epidemiology School of Medicine, University of Nottingham, Nottingham, NG5 1PB UK

**Keywords:** Community-based rehabilitation, Psychosocial intervention, Community mental health services, Schizophrenia, Caregivers, Randomized controlled trial

## Abstract

**Background:**

Schizophrenia is a severe mental health condition with high impact on those affected and their families. Community-based rehabilitation (CBR) is a recommended treatment component for schizophrenia in low- and middle-income countries (LMIC), as it seeks to address complex social, health and economic needs. There is little evidence on the effects of CBR on caregivers of people with schizophrenia. RISE, conducted in Ethiopia, was the first randomised controlled trial of CBR for schizophrenia in a low-income country. In this paper, we extend our previous examination of caregiver impact by (1) investigating the impact of CBR on caregiver stigma and burden, (2) assessing effect modification of outcomes, and (3) determining predictors of caregiver outcomes at 12 months.

**Methods:**

Data are from the cluster-randomised controlled RISE trial, which investigated CBR and facility-based care versus facility-based care alone among 166 people with schizophrenia and 166 linked caregivers in 48 sub-districts in Ethiopia. We analyse the effect of CBR on caregiver stigma, unemployment and burden measured with the WHO Family Interview Schedule-Impact at 6 and 12 months; and caregiver depression, reduction in work due to caregiving and caregiver burden measured with the Involvement Evaluation Questionnaire at 6 months. Logistic and linear regression models adjusted for clustering by sub-district and health centre were used for binary and continuous outcomes respectively. Effect modification by caregiver sex, age, baseline of the outcome, and baseline disability were assessed. Baseline factors associated with caregiver outcomes across the whole cohort at 12 months were investigated using hierarchal regression modelling.

**Results:**

Data were available for 112 caregivers at 6 months (67%), and 149 caregivers at 12 months (90%). There was evidence that CBR was associated with greater tendency to reduce work due to caregiving at 6 months (OR:2.40, 95%CI:1.06–5.45). No evidence of an intervention effect was found on unemployment, depression, stigma or other aspects of caregiver burden. There was no evidence for effect modification. Higher baseline disability was independently associated with greater caregiving burden at 12 months (β:0.26, 95%CI:0.14–0.37).

**Conclusions:**

There appeared to be no positive intervention effect of CBR on caregiver stigma, unemployment and burden in this analysis. Improving the outcomes of caregivers of people with schizophrenia in LMIC requires interventions and research addressing the needs of caregivers, for instance by integrating social and livelihoods interventions.

**Trial registration:**

Clinical Trials.gov Identifier NCT02160249. Registered on 3 June 2014.

**Supplementary Information:**

The online version contains supplementary material available at 10.1186/s12888-025-06651-4.

## Background

Schizophrenia spectrum disorders are severe mental health conditions with a high impact on those affected and their families [1, 2]. These conditions are associated with high levels of disability and poverty, premature mortality, and human rights abuses [3, 4]. Evidence-based treatment for mental health conditions is largely inaccessible in low- and middle-income countries (LMIC), due to a lack of investment in services, a shortage in trained mental health workers and concentration of services in urban areas [5, 6]. In Ethiopia, there is an estimated treatment gap for schizophrenia of 90% [7, 8]. In the past decade, mental health has been integrated into primary care in pilot sites in Ethiopia, which has increased the availability of basic facility-based care (anti-psychotic medication and psychoeducation) for some people with schizophrenia [9, 10]. However, family members are responsible for most care, leading to a heavy financial, social, and emotional burden [2, 11, 12]. These patterns are well established across similar cultural and social contexts [13–15]. Typically women and girls carry out most caregiving responsibilities in Ethiopia [16]. Caregiver burden can be defined as “the level of multifaceted strain perceived by the caregiver from caring for a family member and/or loved one over time” [17]. Caregivers of people with schizophrenia in Ethiopia also experience high levels of stigma [18], spanning problems of knowledge (ignorance), attitudes (prejudice) and behaviour (discrimination) relating to mental illness [19]. Stigma experienced by families may lead to them hiding their relatives and therefore withdrawal from treatment, but also to isolation of the caregivers themselves [20, 21]. Cultural beliefs, such as mental illness being the result of supernatural punishment, may contribute further to the stigma experienced by people with schizophrenia and their families [21].

Community-based psychosocial interventions for schizophrenia can reduce symptom severity and improve functioning of people with schizophrenia in LMIC, yet access to such services is extremely limited [22]. The World Health Organization (WHO) recommends Community Based Rehabilitation (CBR) as a component of the treatment of people with schizophrenia in LMIC [23]. CBR is an approach aimed at improving the quality of life of people with disabilities by involving not only the person with the disability, but also families and communities [24]. It can be delivered by lay workers, making it suitable for low-resource settings. Potential elements of CBR are promoting health, education, livelihoods, and social life, as well as a focus on empowerment. Counselling, problem-solving techniques, and community mobilisation are elements that are frequently used in the context of CBR for mental health and psychosocial disabilities [25].

The CBR for people with Schizophrenia in Ethiopia (RISE) cluster-randomised trial found that CBR is effective in reducing disability and symptom severity among people with schizophrenia in a rural district in south-central Ethiopia who had not improved or engaged with primary mental health care services [26] [20]. 

Caregivers are important actors in CBR but there is an evidence gap on the effects of CBR, and other community-based psychosocial interventions, on the experience of caregivers of people with schizophrenia. While recognizing the effects of CBR on people with schizophrenia and their caregivers are likely bidirectional, we hypothesised that improved functioning and symptoms in people with schizophrenia would lead to improvements in caregivers’ experience. This hypothesis is supported by a previous observational study in Ethiopia showing reduced symptoms and longer periods in remission in people with severe mental health conditions was associated with reduced caregiver burden [27]. Furthermore, findings from the RISE trial demonstrated a beneficial intervention effect of CBR on caregiver burden at 12 months, measured by the Involvement Evaluation Questionnaire (IEQ), in the domains of tension and worrying. There was no evidence of an effect on depression or reduction in work due to caregiving at 12 months [26], but more extensive analysis of outcomes for caregivers has not yet been done

 In this paper we explore the effect of CBR on the additional pre-specified exploratory outcomes of caregiver stigma, unemployment and burden measured with the WHO Family Interview Schedule-Impact at 6 and 12 months; and caregiver depression, reduction in work due to caregiving and caregiver burden measured with the IEQ at 6 months, among participants in the RISE trial. Secondary aims were to assess (i) effect modification of any CBR effect by participant and caregiver characteristics and (ii) the determinants of caregiver outcomes across the whole cohort.

## Methods

### Study design and participants

This study uses data on caregiver outcomes from the parallel-arm cluster-randomised controlled RISE trial, which compared CBR plus facility-based care to facility-based care alone [26, 28]. The RISE trial was conducted in Sodo District, Gurage Zone, Southern Nations, Nationalities and People’s Region, Ethiopia. The study protocol and main trial results were previously published [26, 28]. Sodo District has an estimated total population of 170,000 people in 58 sub-districts [29]. Most of the population live in remote rural areas [29]. The district has high levels of poverty, and the main economic activity is subsistence farming. Primary care is delivered by health officers and nurses at one primary hospital and seven health centres, each catering to a mean of six sub-districts [29]. 

The unit of randomisation in the trial was sub-district, and a total of 54 sub-districts were allocated using minimization in a 1:1 ratio to the intervention arm (facility-based care plus CBR) and the control arm (facility-based care alone). Randomization and masking have been described previously [26]. Trial participants were individuals with a diagnosed schizophrenia spectrum disorder, recruited from the PRIME (PRogramme for Improving Mental healthcarE) cohort study, which had already implemented facility-based mental health care in primary health centres for 6 months in the area [30]. Diagnoses was assessed by trained psychiatric nurses using the Operational Criteria for Research (OPCRIT) diagnostic interview (which applies Diagnostic and Statistical Manual of Mental Disorders fourth edition [DSM-IV] criteria) [31].Only participants with high levels of disability defined by either a Brief Psychiatric Rating Scale-Expanded (BPRSE) score ≥ 52, proxy or self-rated 36-item WHO Disability Assessment Schedule (WHODAS) 2.0 score ≥ 35, continuous illness lasting 6 months, symptomatic for ≥ 3 of last 6 months, or Clinical Global Impression (CGI) severity score ≥ 3 after 6 months access to facility-based care were included in the RISE trial [32–36]. Written informed consent was taken from potential participants. A nurse determined whether the person with schizophrenia had the capacity to give informed consent. If they were deemed to not have capacity, a caregiver gave consent for them, but assent was still sought from the person with schizophrenia. Additionally, if the person with schizophrenia regained capacity in the process of the study, their consent was sought again at that point. Participants were followed up at 6 months (midline) and 12 months (endline) after recruitment. Data were collected in the health centre or at participants’ homes by trained lay data collectors and by a trained psychiatric nurse.

For each trial participant, one primary caregiver was identified. Caregivers were eligible if they were aged 18 years or older and provided regular support to the person with schizophrenia. This would be regular in-person contact and support in different forms such as providing meals and/or shelter, financial support, and assisting to go to health facility. The caregiver can be a spouse, parent, sibling, other relative or friends. If the original caregiver was unavailable at endline, caregiver-reported data were collected from a different caregiver meeting the criteria. As this could have affected the reliability of the results, a sensitivity analysis was done excluding records that had a different caregiver at endline.

### Procedures

The interventions have been described in detail previously [26, 28]. All participants had access to facility-based care, which is a stepped care model in which most care is delivered in primary care. It primarily comprised prescription of anti-psychotic medication and psychoeducation by nurses and health officers trained in the WHO mental health Gap Action Programme-Intervention Guide (mhGAP-IG) [23].

Intervention clusters additionally received a CBR intervention which had been shown to be acceptable and feasible in the rural Ethiopian context [9, 37, 38]. CBR workers were lay people from the local area with at least ten years of education but no prior experience in delivering mental healthcare. They received five weeks initial training in CBR delivery, guided by a manual, including basic counselling and problem-solving techniques [38]. Training was split between classroom teaching and fieldwork.

CBR was delivered by 11 CBR workers, each supporting a median of seven participants and their families (range 4–11). CBR visits lasted 30–90 min and took place at the participants’ home. Caregivers were requested to participate in every visit. The intervention emphasized human rights, social inclusion and personal recovery. Visits were every 1–2 weeks for the first three months, then every 2 weeks for the next five months, and monthly for the final four months. Two supervisors oversaw the home visit content and frequency. Core modules covered understanding schizophrenia, access to health services, crisis management and human rights. Optional modules included adherence support, family intervention, support returning to work and social activities, and dealing with stigma. Caregivers were intimately involved in CBR, for example, contributing to goal setting, reminding their relative to take medication, accompanying them to community activities, and reducing unhelpful communication (such as excessive criticism) in the home. The aim was to empower caregivers to keep up the positive effects of CBR after the intervention had terminated. In the CBR worker training manual there were dedicated chapters on ‘Impact of schizophrenia on the family’ and ‘Being aware of the caregiver’s needs’. CBR workers were trained to be aware of potential distress in caregivers and to propose solutions such as speaking with, or sharing the practical or financial burden with, relatives and neighbours; and attending the health centre for mental health support. CBR workers met with community members to mobilise resources for individual participants, conducted public awareness-raising meetings and ran family support groups. These groups were intended as places for caregivers to gain mutual emotional support.

### Instruments and measurements

**Fig. 1:**
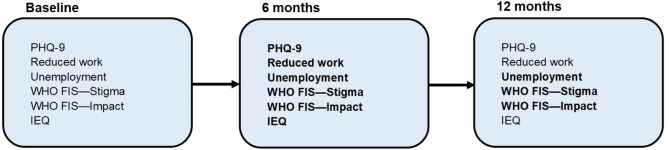
Flowchart of caregiver outcomes collected at different timepoints Outcomes measurements highlighted in bold are newly analysed in this paper

The primary trial outcome was participant disability (proxy-reported 36-item WHODAS) at 12 months [28]. The rationale for a proxy-reported measure was that reporting can vary among people with schizophrenia spectrum disorders depending on their mental state [26]. The present paper focuses on the following pre-specified exploratory trial outcomes in caregivers: caregiver employment at 6 and 12 months, caregiver stigma at 6 and 12 months (WHO Family Interview Schedule (WHO FIS)- Stigma total score), caregiver burden measured with WHO FIS- Impact at 6 and 12 months, caregiver burden measured with IEQ at 6 months, reduction in work due to caregiving at 6 months (binary question) and caregiver depression at 6 months (PHQ-9 ≥ 5 and total score) [39–42]. There is some overlap between the WHO FIS-Impact and IEQ but both also cover distinct domains. The WHO FIS-Impact assesses impact of caring on social life, work, financial circumstances and family strain. The IEQ assesses the degree of encouragement and care caregivers give to their family member (‘urging’ and ‘supervision’ domains), and the emotional burden on the caregiver and family (‘worrying’ and ‘tension’ domains). PHQ-9 had been validated in Ethiopia [43, 44]. WHO FIS has previously been used in Ethiopia, but IEQ has not [2, 18, 45]. We measured caregiver burden in multiple ways be able to validate potential effects on burden using multiple measures of this concept. The appendix (A1) includes details on the validity of these measures. In the Ethiopian setting, a PHQ-9 score *≥* 5 indicates possible major depressive disorder [44]. We have previously presented the intervention effect on IEQ, caregiver depression and reduced work due to caring at 12 months [26]; the IEQ analysis was repeated in order to provide a foundation for the analysis of effect modification and determinants of caregiver outcomes. Figure 1 shows a schematic overview of caregiver outcomes assessed at each timepoint.

### Sample size

Of the 166 caregivers recruited into the RISE trial, 149 (89.8%) were seen within the pre-specified window for analysis (+/- 10 weeks of endline) at 12 months. This provided 86% power to detect a standardised mean difference (SMD) of 0.5 for continuous outcomes. We assumed Type 1 error of 5%, and used the intra-cluster correlation (ICC) of 0.02 from the observed data. The ICC is a measure of the relatedness of clustered data [46]. Similarly, there was 80% power to detect a difference in proportions of e.g. 50% vs. 28% with the same assumptions. For the 6 month outcomes, 112 (67.4%) caregivers were seen within the pre-specified window for analysis (+/- 10 weeks of endline). This gave 80% power to detect an SMD of 0.56 with a Type 1 error of 5%.

### Statistical analysis

Statistical analysis was done with Stata (version 16). The appendix (A2) includes a table of the coding of all variables used in this report. To assess differences between treatment arms at baseline, cross-tabulations for categorical variables and means and medians for continuous variables were used. Baseline caregiver and participant characteristics were compared between those with and without complete 6 and 12-month outcome data. We used random-effects linear and logistic regression for continuous and binary outcomes respectively. We adjusted for clustering by sub-district as a random effect and health center as a fixed effect. Missing data were recoded as a separate category for exposure variables so no observations would be dropped.

The effectiveness analyses used intention-to-treat principles. For all binary outcomes, Generalized Estimating Equation (GEE) models were used instead of random-effects models. This is because the difference between the quadrature points was > 0.01% in the random-effects models which is an indication the random-effects model is unreliable [47]. GEE is a method to account for correlated data and is suitable for studies with a clustered design. For continuous variables, a linear regression random-effects model was fitted. We included sub-district cluster as a random effect and health centre as a fixed effect. Minimally-adjusted analyses were first fitted, including only the baseline score of the outcome of interest and the clustering variables. As minimally-adjusted analysis only takes into account a minimum of variables, next, fully-adjusted models, which are models adjusting for all possible confounders, were fitted. These models additionally adjusted for baseline WHODAS score, variables associated with missingness, and variables unbalanced at baseline [48, 49]. We created a final model through a backwards modelling strategy. This meant all covariates that did not change the effect estimate by 10% or more when taken out of the model were removed. If the model had computational issues based on collinearity of the included variables one of the variables was dropped.

Effect-modification of intervention arm with caregiver’s age and sex, baseline WHODAS score (dichotomized with a cut-off of 40), and baseline of the outcome of interest was assessed for those outcomes associated with CBR. We did this by fitting an interaction term with the effect-modifier of interest and intervention arm in those final models. Validity checks and sensitivity analyses were done. This was done using a log-transformation on the outcome variable when that variable was not normally distributed. Other sensitivity analyses included: (i) outcome data that were collected outside the specified +/- 10-week window, (ii) excluding those records that related to a different caregiver at that timepoint than at baseline, (iii) multiple imputation and (iv) re-running the models without missingness coded as a category for the covariates. See appendix A3 for details of analyses.

**Fig. 2:**
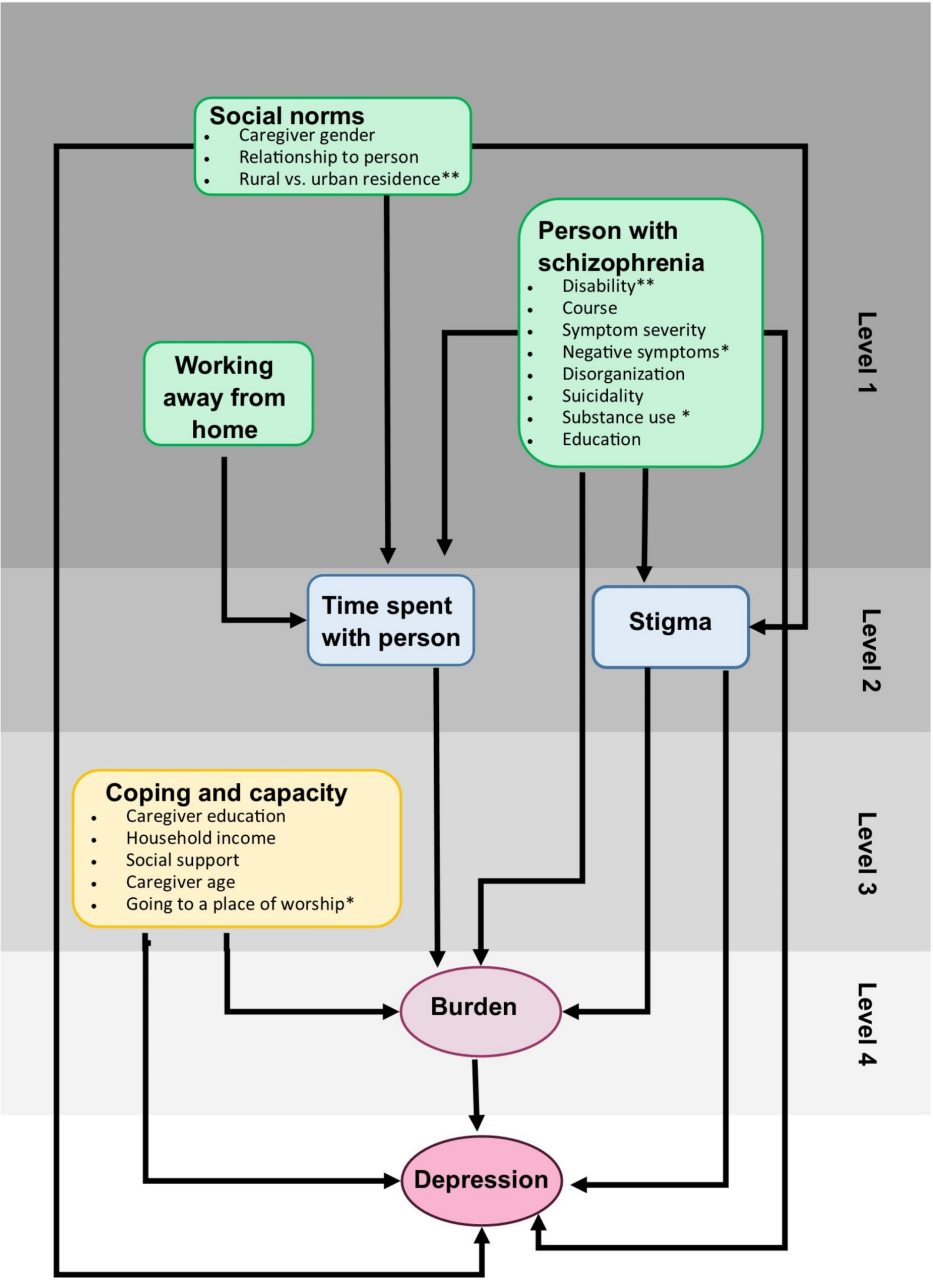
Conceptual framework for caregiver outcomes *No variable was available in the dataset for these factors. Alcohol use disorder variable was used for substance use disorder ** These variables were not identified directly from literature, but added by the authors based on experience of evidence of indirect association in the literature

We examined factors associated with caregiver burden (IEQ total score) and depression (PHQ9 *≥* 5) at 12 months using a hierarchical conceptual framework [50] based on the peer-reviewed literature (appendix A4, Fig. 2) [2, 11, 18, 21, 27, 51, 52]. Based on the conceptual framework, we identified the following levels for the hierarchical analysis:

Level 1: Factors relating to the person with schizophrenia, social norms, and caregiver working away from home (most distal).

Level 2: Time spent with person with schizophrenia and stigma.

Level 3: Caregiver coping and capacity.

Level 4: Burden (most proximal).

Univariable analyses were conducted to assess the association between each exposure variable with depression and burden separately. We used GEE logistic regression models for depression and random effects linear regression for burden. Factors that had a Wald-test p-value lower than 0.1 in the univariable analysis were included as covariates for the multivariable analysis. First, models were run including all the Level 1 variables that had a *p* < 0.1 in the univariate analysis (model 1). Variables were retained if they were independently associated with the outcome (*p* < 0.1) (model 1b). The subsequent levels were then added stepwise, to indicate whether variables in the distant levels are mediated through variables in the proximate levels.

### Ethical approval

The study was approved by the LSHTM Research Ethics Committee (reference 0735-2), the Addis Ababa University College of Health Sciences Institutional Review Board (reference 083/13/Psy), and the Ethiopian National Research Ethics Review Committee (reference 310/048/2015).

## Results

### Participant characteristics

Trial participants and caregivers were enrolled between September 16, 2015 and March 11, 2016. Of the 54 available sub-districts, 27 were randomised to the intervention arm and 27 to the control arm. A total of 294 potential participants were pre-screened, of whom 91 were excluded. A further 37 individuals were not enrolled. Of these, 22 participants (10·8%) did not meet the inclusion criteria, one participant and six caregivers declined (3·4%) and eight participants were excluded due to already reaching sufficient numbers in the cluster (3·9%). Three sub-districts were excluded at each of the pre-screening and enrolment stages. Hence of 54 potential sub-districts for inclusion, 48 were included. Twenty-four sub-districts (79 participants and linked caregivers) were assigned to the intervention arm and 24 sub-districts (87 participants and linked caregivers) were assigned to the control arm.

The 166 caregivers had a mean age of 41 years (SD = 14.7). Approximately half were male (*n* = 77; 46.4%), and half were aged > 35 years (*n* = 93; 56.0%). Two thirds of caregivers were married (*n* = 111; 66.9%), and almost all were either a first degree relative or a spouse of the person with schizophrenia (*N* = 149; 89.8%). The majority had no education (*N* = 96; 57.8%), whilst 11.4% (*N* = 19) had more than 8 years of education. About one third (*N* = 63; 38.0%) were illiterate. Most caregivers had reduced their work due to the caring burden (*N* = 134; 80.7%) and 44.0% of caregivers had depression (PHQ ≥ 5) (*N* = 73). 11 (6.6%) had missing data on at least one baseline characteristic.

Caregiver depression (PHQ ≥ 5) and caregiver employment were considered unbalanced by arm at baseline (Table 1), along with several participant characteristics (sex, education level, socioeconomic status, employment status, the level of social support, and occupation; reported previously [26]).

**Table 1:** Baseline characteristics of caregivers and caregiver outcomes at baseline

	Intervention	Control
Sex (N [%])*		
Male	33 (41.8%)	44 (50.6%)
Female	42 (53.2%)	36 (41.4%)
Missing	4 (5.1%)	7 (8.1%)
Age group (N [%])*		
< 25	15 (19.0%)	14 (16.1%)
5–35	18 (22.8%)	15 (17.2%)
35>	42 (53.2%)	51 (58.6%)
Missing	4 (5.1%)	7 (8.1%)
Marital status (N [%])		
Single	14 (17.7%)	12 (13.8%)
Married	49 (62.0%)	62 (71.3%)
Divorced or Widowed	16 (20.3%)	12 (13.8%)
Missing	0	1 (1.1%)
Education in years (N [%])*		
0	46 (58.2%)	50 (57.5%)
1–8	17 (21.5%)	23 (26.4%)
9–15	12 (15.2%)	7 (8.1%)
Missing	4 (5.1%)	7 (8.1%)
Literacy (N [%]) *		
Illiterate	32 (40.5%)	31 (35.6%)
Can read and write	14 (17.7%)	19 (21.8%)
Formal education	29 (36.7%)	30 (34.5%)
Missing	7 (8.1%)	4 (5.1%)
Relationship to person with schizophrenia (N [%])		
Parent	18 (22.8%)	27 (31.0%)
Sibling	14 (17.7%)	22 (25.3%)
Child	21 (26.6%)	11 (12.6%)
Spouse	16 (20.3%)	18 (20.7%)
Other	10 (12.6%)	9 (10.4%)
**Outcomes at baseline**		
Reduced work due to caring		
No	15 (19.0%)	17 (19.5%)
Yes	64 (81.0%)	70 (80.5%)
Caregiver unemployment (N [%])		
Employed	43 (54.4%)	55 (63.2%)
unemployed	36 (45.6%)	32 (36.8%)
Depression (PHQ > 4) (N [%])		
No	36 (45.6%)	57 (65.5%)
Yes	43 (54.4%)	30 (34.5%)
PHQ total (median [IQR])	5 (2–8)	3 (2–6)
IEQ		
Urging (median [IQR])	14 (9–20)	16 (10–20)
Supervision (median [IQR])	9 (4–13)	8 (4–12)
Worrying (median [IQR])	13 (6–17)	11 (8–15)
Tension (median [IQR])	8 (4–11)	7 (4–11)
Total (median [IQR])	41 (26–53)	41 (29–50)
FIS		
Total stigma (median [IQR])	10 (5–18)	8 (3–17)
Total impact (median [IQR])	4 (0–7)	3 (1–7)

*These were collected at PRIME baseline

In each arm, 27 caregivers (31.0% for the control arm and 34.2% for the intervention arm) did not complete the outcome assessment at 6 months. At 12 months, 11 (12.6%) caregivers in the control arm and 6 (7.6%) in the intervention arm did not complete the assessment. Characteristics associated with missingness at 12 months were urban residence (*p* = 0.06), higher socioeconomic status (*p* = 0.02), higher caregiver burden (IEQ score) (*p* = 0.03), and shorter illness duration (*p* = 0.04), relapse in preceding 6 months (*p* = 0.04), older age (*p* = 0.05), male sex (*p* = 0.06) amongst participants with schizophrenia. Female caregivers (*p* = 0.07) with longer travel time to the facility (*p* = 0.07) were more likely to have missing data at 6 months.

### Effect of CBR on caregiver outcomes

Table 2 shows the results of the minimally adjusted and final models respectively for caregiver outcomes. In the final model there was evidence that caregivers who were randomised to the CBR arm were more likely to have reduced their work due to undertaking caring activities at 6 months compared to those in the control arm (aOR = 2.40, 95%CI:1.06–5.45, *p* = 0.04) as highlighted in bold in the table. There was no evidence of an intervention effect for other outcomes in the final model or for any outcome in the minimally adjusted models. The sensitivity analyses were broadly consistent with the main analysis (appendix A5 and A6).

**Table 2:** Caregiver outcomes at 6 and 12 months

Outcome	Control *N* (%) or mean (SD)	Intervention *N* (%) or mean (SD)	Minimally adjusted mean difference or odds ratio (95%CI)^a^	*p*- value	Final model mean difference or odds ratio (95% CI)^b^	*p*-value	Standardized mean difference (95% CI)
**6 months (***N* = **112)**							
**Reduced work due to caring**	45 (75.0%)	44 (84.6%)	OR 1.92 (0.79,4.68)	0.15	**OR 2.40 (1.06**,**5.45)**	**0.04**	-
**Caregiver unemployment**	22 (36.7%)	28 (53.9%)	OR 2.16 (0.56,8.38)	0.26	OR 2.09 (0.55,7.95)^ijn^	0.28	-
**PHQ**							
** Depression (> 4)** ^e^	21 (35.0%)	20 (38.5%)	OR 1.26 (0.46,3.47)	0.65	OR 1.58 (0.52,4.84)^iln^	0.42	-
** Total score**	3.52 (2.91)	4.04 (3.51)	0.14 (-1.50,1.78)	0.87	0.61 (-0.87,2.09)^ln^	0.42	0.19 (-0.18,0.56)
**FIS**							
** Total stigma**	10.2 (8.84)	9.96 (9.17)	0.38(-3.46,4.21)	0.85	0.54 (-4.26,5.33)^ceijlqn^	0.83	0.06 (-0.31,0.43)
** Total impact**	4.23 (4.01)	3.53 (3.63)	-1.01 (-2.95,0.94)	0.31	-0.69 (-2.79-1.41)^cejln^	0.52	-0.18 (-0.55,0.19)
**IEQ**							
** Urging**	14.68 (7.88)	13.46 (6.57)	-0.15 (-3.13,2.84)	0.92	-0.76(-4.09,2.58)^ehijln^	0.65	-0.10 (-0.48,0.27)
** Supervision**	7.67(6.58)	7.54 (5.96)	-0.88 (-3.58,1.82)	0.52	-1.71 (-4.94,1.53)^ejln^	0.30	-0.27 (-0.64,0.10)
** Worrying**	10.28 (6.86)	8.68 (5.86)	-1.12 (-3.60,1.35)	0.37	-1.42 (-3.94,1.10)^el^	0.27	-0.22 (-0.59,0.15)
** Tension**	6.3 (5.64)	6.96 (6.84)	0.98 (-1.83,3.79)	0.49	1.07 (-1.83,3.97)^eln^	0.47	0.17 (-0.20,0.54)
** Total**	36.47 (19.84)	34.22 (18.41)	-0.33 (-9.29, 8.62)	0.94	-1.67 (-11.65,8.31)^ecijm^	0.74	-0.09 (-0.46,0.28)
**12 months (***N* = **149**)							
**Caregiver unemployment**	43 (56.6%)	40 (54.8%)	OR 1.09 (0.60,1.97)	0.78	OR 0.72 (0.35,1.50)^cdefk^	0.38	-
**FIS**							
** Total stigma**	10.89 (10.15)	9.05 (9.30)	-2.52 (-6.08,1.03)	0.16	-2.57 (-6.28,1.13)^c^	0.17	-0.27 (-0.60,0.05)
** Total impact**	4.21 (3.69)	5.25 (3.93)	1.17 (-0.31,2.65)	0.12	0.98 (-0.55-2.52)^cdfj^	0.21	0.26 (-0.06,0.58)

Socio-economic status, urban vs. rural residence, caregiver sex, illness duration and travel time to the facility were coded to have missingness as a category

^a^ Adjusted for sub-district (cluster) as random effect and health centre and baseline score of outcome as fixed effects

^b^ All are adjusted for sub-district (cluster) as random effect and health centre, baseline score of outcome, and baseline WHODAS score as fixed effects in addition to the variables that were found to have a confounding effect

^c^ Adjusted for socioeconomic status

^d^ Adjusted for place of residence

^e^ Adjusted for baseline depression

^f^ Adjusted for disease course prior to study

^g^ Adjusted for baseline total IEQ score at baseline

^h^ Adjusted for the patients sex

^i^ Adjusted for the patients employment status

^j^ Adjusted for social support

^k^ Adjusted for the patients age

^l^ Adjusted for the caregivers sex

^m^ Adjusted for the caregivers employment

^n^ Adjusted for travel time to the facility

There was no evidence of effect modification by caregiver’s age and sex, baseline WHODAS score and baseline worrying (dichotomized with a cut-off of 10), tension (dichotomized with a cut-off of 10), or reduced work.

### Factors associated with caregiver outcomes

Disability, disorganized symptoms, lower caregivers’ education, being a first-degree family member or spouse, the caregiver not going out of the home to work, stigma, and time spent with the person with schizophrenia met the *p* < 0.10 threshold in the univariable analysis related to burden (see appendix A7). The result of the hierarchical linear regression models for burden at 12 months are shown in Table 3. Higher levels of disability were consistently associated with higher caregiver burden in all models (*p* < 0.001). First-degree relatives and spouses reported higher levels of burden than more distant relatives or unrelated caregivers. Caregiver stigma was also independently associated with greater burden.

**Table 3:** Hierarchical linear regression models for burden measured by total IEQ score at 12 months

	1 (*N* = 147)		1b (*N* = 149)		2 (*N* = 149)		2b (*N* = 149)		3 (*N* = 138)		3b (*N* = 149)	
	β (95%-CI)	*p*-value	β (95%-CI)	*p*-value	β (95%-CI)	*p*-value	β (95%-CI)	*p*-value	β (95%-CI)	*p*-value	β (95%-CI)	*p*-value
**Level 1**												
**WHODAS**	0.26 (0.14–0.38)	< 0.001	0.29 (0.18–0.40)	< 0.001	0.26 (0.15–0.37)	< 0.001	0.26 (0.15–0.37)	< 0.001	0.26 (0.14–0.38)	< 0.001	0.26 (0.14–0.37)	< 0.001
**Disorganisation**		0.15										
Absent	0											
Present	4.93 (-1.75 -11.61											
**Relationship**		0.003		0.01		0.03		0.01		0.05		0.01
1st degree/spouse	0		0		0		0		0		0	
Other relationship	-13.78 (-23.02- -4.54)		-13.97 (-22.31 - -3.82)		-10.26 (-19.74 - -0.77)		-11.92 (-21.12- -2.71)		-9.61 (-19.29-0.05)		-11.89 (-21.19- -2.59)	
**Works away from home**		0.02		0.03		0.05		0.05		0.24		
No	0		0		0		0		0			
Yes	-6.23 (-11.59- − 0.87)		-6.03 (-11.36- -0.70)		-5.33 (-10.63 - -0.03)		-5.31 (-10.63-0.00)		-3.45 (-9.21-2.30)			
**Level 2**												
**Stigma**					0.29 (-0.02–0.59)	0.06	0.31 (0.01–0.61)	0.04	0.30 (-0.01-0.61)	0.06	0.35 (0.05–0.65)	0.02
**Time spent**						0.17						
< 32 h/week					0							
> 32 h/week					4.84 (-2.14-11.82)							
**Level 3**												
**Caregiver years of education**										0.16		
0									0			
1–8									-2.06 (-8.62-4.50)			
9–15									-9.06 (-18.34-0.22)			

All models are adjusted for sub-district (cluster) as random effect and health centre as a fixed effect. All other covariates in the model are measured at baseline. The model number represents the level of variables included, and the b-versions are those with only the variables independently associated with the outcome (*p* < 0.10)

Disability, social support, caregiver gender, residence, relationship to the person with schizophrenia, caregiving burden, time spent with person with schizophrenia, and stigma met the *p* < 0.10 threshold in the univariable analysis relating to depression (see appendix A8). Table 4 shows the results for the hierarchical logistic regression models of depression at 12 months. Disability was initially associated with depression, but not after adjusting for caregiving burden (*p* = 0.89) suggesting this was largely mediated through burden, assuming the framework is correct. Living in a rural area was independently associated with higher depression (OR = 8.56, 95%-CI:1.33–44.08). Adult children of the person with schizophrenia had the highest odds of depression, and siblings and other relatives had the lowest. Stigma and burden were also independently associated with higher odds of depression (OR = 1.04, 95%-CI:1.00-1.08; OR = 1.03, 95%-CI:1.01–1.05 respectively for a unit increase in score).

**Table 4:** Hierarchical logistic regression models for depression measured by PHQ > 4 at 12 months

	1		1b		2		2b		3		3b		4		4b	
	OR (95%-CI)	*p*-value	OR (95%-CI)	*p*-value	OR (95%-CI)	*p*-value	OR (95%-CI)	*p*-value	OR (95%-CI)	*p*-value	OR (95%-CI)	*p*-value	OR (95%-CI)	*p*-value	OR (95%-CI)	*p*-value
**Level 1**																
**WHODAS**	1.01 (1.00-1.03)	0.05	1.01 (1.00-1.03)	0.05	1.01 (1.00-1.03)	0.10	1.01 (1.00-1.03)	0.10	1.01 (1.00-1.03)	0.06	1.01 (1.00-1.03)	0.10	1.00 (0.98–1.02)	0.89		
**Sex**		0.56														
Male	1															
Female	1.25 (0.60–2.60)															
**Place of residence**		0.02		0.02		0.03		0.03		0.02		0.03		0.02		0.02
Urban	1		1		1		1		1		1		1		1	
Rural	8.90 (1.48–53.65)		8.61 (1.41–52.47)		7.89 (1.24–50.08)		8.02 (1.25–51.41)		9.99 (1.52–65.69)		8.02 (1.25–51.41)		8.59 (1.32–56.02)		8.56 (1.33–55.08)	
**Relationship**		0.01		0.01		0.01		0.01		0.01		0.01		0.01		0.01
Parent	1		1		1		1		1		1		1		1	
Sibling	0.13 (0.03–0.58)		0.12 (0.03–0.55)		0.14 (0.03–0.71)		0.12 (0.02–0.61)		0.11 (0.02–0.57)		0.12 (0.02–0.61)		0.13 (0.02–0.67)		0.12 (0.02–0.67)	
Child	1.58 (0.54–4.64)		1.64 (0.57–4.68)		1.84 (0.61–5.52)		1.69 (0.59–4.86)		1.71 (0.57–5.14)		1.69 (0.59–4.86)		1.94 (0.65–5.75)		1.95 (0.66–5.78)	
Spouse	0.52 (0.16–1.71)		0.52 (0.16–1.72)		0.52 (0.16–1.73)		0.54 (0.17–1.74)		0.58 (0.16–2.06)		0.54 (0.17–1.74)		0.51 (0.15–1.75)		0.52 (0.15–1.78)	
Other	0.14 (0.02–0.83)		0.12 (0.02–0.77)		0.20 (0.03–1.24)		0.15 (0.02–1.01)		0.13 (0.02–0.91)		0.15 (0.02–1.01)		0.24 (0.04–1.48)		0.24 (0.04–1.42)	
**Level 2**																
**Stigma**					1.05 (1.00-1.09)	0.04	1.05 (1.00-1.09)	0.03	1.05 (1.01–1.10)	0.02	1.05 (1.00-1.09)	0.03	1.04 (1.00-1.09)	0.06	1.04 (1.00-1.08)	0.06
**Time**						0.23										
< 32 h/week					1											
> 32 h/week					1.85 (0.67–5.07)											
**Level 3**																
**Social support**										0.32						
Strong/intermediate									1							
Poor									0.65 (0.27–1.53)							
**Level 4**																
**Burden**													1.03 (1.00-1.06)	0.02	1.03 (1.01–1.05)	0.01

All models are adjusted for sub-district (cluster) as random effect and health centre. All other covariates in the model are measured at baseline. *N* = 137 for all models. The model number represents the level of variables included, and the b-versions are those with only the variables independently associated with the outcome (*p* < 0.10)

## Discussion

This analysis of exploratory data from the RISE trial looked at depression, burden, employment, reduction in work due to caring and stigma among caregivers of people with schizophrenia in Ethiopia. RISE is the first randomised trial of CBR for people with schizophrenia in a low-income country, which means our results offer new insights into caregiver outcomes in such settings. With a few exceptions [53–55], most previous evaluations of psychosocial interventions for people with schizophrenia in LMIC have not assessed the impact on caregivers and few, to our knowledge, have found a positive effect. We have previously demonstrated that, there was evidence that CBR is effective in reducing worrying and tension in caregivers at 12 months [26]. In the current analysis, CBR appeared to increase caregivers’ need to reduce their work due to caring at 6 months. This may be because caregivers were influenced by CBR to undertake more frequent or intensive caring activities, such as accompanying their relative to the health centre or to social activities. Alternatively, physically attending CBR sessions may have meant caregivers had less time to work, thereby indicating a possible negative effect of CBR. The fact that this effect was seen only at 6 months, when sessions were more frequent, but not at 12 months [26], when session frequency was monthly, supports this hypothesis. We found in the RISE pilot study that CBR participation was sometimes less disruptive for female compared to male participants because women could continue their usual work (for example, preparing food or handicrafts) whilst speaking with the CBR worker [9]. However men’s typical work roles, for example, tending to livestock, were less compatible. Given poverty was the most pressing concern expressed by families affected by schizophrenia, the finding that CBR had reduced the ability of caregivers to work at 6 months is worrisome [29, 37]. Future research on psychosocial interventions in LMIC should this consider how to mitigate this possible negative effect.

Our finding of no intervention effect on caregiver stigma, depression or employment is likely to be because CBR does not adequately address those specific aspects of caregivers’ experiences. For example, there were no psychological or livelihoods interventions targeting caregivers embedded within CBR. We also previously found CBR to be ineffective in reducing discrimination or food insecurity, or improving employment or work functioning in people with schizophrenia [26, 56]. CBR is therefore unlikely to decrease poverty levels. Previous research in the study district has found high levels of food insecurity amongst families affected by schizophrenia [57]. Our formative work identified poverty as the most pressing need experienced by this group [37]; CBR’s limited ability to affect this underpinning concern and the apparent increased need to reduce work in at 6 months may explain the absence of impact on several aspects of caregiver burden. Furthermore, only 7/24 (30%) of sub-districts ran a family support group and 3/24 (13%) subdistricts had only 1 or 2 meetings [26]. The absence of opportunities for mutual support amongst caregivers may have limited the impacts of CBR on caregiver outcomes. Additionally, there might have been cultural factors affecting burden that the intervention addressed insufficiently. Our intervention development work found all stakeholders, including caregivers and individuals with schizophrenia, found CBR an acceptable intervention, the CBR workers were from the local area, and the intervention was not dismissive of traditional healing practices such as visiting holy water sites, but there could have been cultural barriers specific to caregivers that remained unaddressed [37]. A possible improvement of CBR could be to add more modules focussed on caregivers or link caregivers to social and livelihood interventions to help target poverty.

The lack of effect on several caregiver outcomes reflects findings from the COPSI trial of community-based care for people with schizophrenia in India, which also did not demonstrate effects of CBR on caregiver burden or stigma [55]. The intervention used in COPSI did not include community mobilisation which CBR did include [37]. However, similar to the qualitative results from the COPSI trial, qualitative data from the RISE pilot study suggested that caregivers worried less and felt less need to supervise their relative, giving them more time for daily tasks [9]. It is therefore also possible that the instruments used to measure caregiver outcomes were not sensitive enough to change during the intervention period.

There was an independent association between greater baseline disability and increased burden at 12 months. This aligns with previous studies indicating that greater functional impairment is associated with higher caregiver burden [27, 51, 58]. However, we found weaker evidence for an association between baseline disability and depression and, assuming the hierarchical framework is correct, this relationship appeared to be fully mediated through burden. This suggests that it is the high burden caused by greater disability that leads to depression in caregivers. However, it is possible the model does not reflect the likely more complex and bidirectional relationship between burden and depression well enough, as it could also be that caregivers experiencing depressive symptoms are inclined to report higher burden. There was evidence that the relationship the caregiver had to the person with schizophrenia was important. First degree relatives and spouses experienced more burden than more distantly related or unrelated caregivers, and this relationship appeared to be partly mediated by stigma. This suggests that the stigma associated with schizophrenia was felt more by people that are more closely related. We also found that caregivers who were children and parents of the person with schizophrenia experienced the most depression. Whilst a previous Ethiopian study did not find an association between type of relative and caregiver depression [11], a further study in Ethiopia did find other severe intergenerational impacts in families of people with severe mental conditions, such as an increased risk of food insecurity, lower school attendance and even an increased risk of death [59].

### Strengths and limitations

This study had a few strengths. One is the use of multiple sensitivity analyses, which confirmed the validity of the analyses. The use of longitudinal data for the exploratory analysis of factors influencing caregiver outcomes, in contrast to previous cross-sectional studies [60], made it less likely that associations with the outcomes are due to reverse causality.

This study also had a number of limitations. Firstly, not all instruments used to measure the outcome in caregivers were validated for the Ethiopian context. The missing data at the 6-month assessment might have introduced selection bias, which might not have been fully mediated by including variables associated with missingness in the analysis. However, loss to follow-up was low at the 12-month assessment. While results of sensitivity analyses using multiple imputation and re-running the models without missingness coded as a category for the covariates showed broadly consistent results, missing data could still have affected the results. Another issue was that the caregiver that attended the 6 month or 12-month assessment was not always the same person that was interviewed at baseline. This approach could conceivably have under- or over-estimated the observed effect sizes. However sensitivity analysis showed that omitting those caregivers did not change the results of the main analysis. However, we did not run sensitivity analysis for the hierarchical analysis of factors associated with caregiver outcomes, so the effect of different caregivers at baseline and endline on that are unclear. The participants and caregivers were only followed up for 12 months and this short follow-up period is a limitation for assessing long term treatment effect and sustained impact. There was also a lack of power to detect smaller changes for most of the outcomes and the effect modification analysis. There were also computational and reliability issues running the random effects logistic regression models, meaning ORs are based on GEE and thus represent population average rather than cluster-specific ORs [61]. As population average ORs tend to be closer to 1, this might have under-estimated the true effects, or contributed to not detecting evidence for an association with binary outcomes.

There might be factors influencing caregiver outcomes that we were unaware of or did not have data for and thus failed to include in the conceptual framework. Additionally the conceptual framework could have been improved by involving caregivers in the development. Using a p-value based threshold to include factors in the model also means that there is likely to be insufficient correction for many of the confounding factors, as some variables that did not meet the threshold might still have confounded the relationship between the outcomes and other variables in the model. This method was chosen as it allowed for modelling many different parameters on the four hierarchical levels, while preventing having too many parameters in the model [50].

### Implications

We suggest the individual with schizophrenia together with their family should be the unit for delivery of psychosocial support in LMIC for three reasons. First, earlier findings suggest caregivers can play an essential role in the rehabilitation of people with schizophrenia particularly in LMIC like Ethiopia, where access to mental health services is limited and care falls almost entirely on caregivers [2, 7, 9]. Second, caregivers also have the right to health and their needs should be considered. Addressing caregivers’ needs may conceivably mean they are better equipped to support their relative, including encouraging engagement with health services and interventions such as CBR. Whilst psychosocial interventions directly targeting family members of people with schizophrenia are less successful in improving distress, depression or burden, they are effective in improving knowledge and coping [62, 63]. Yet in settings with limited mental health resources, stand-alone caregiver interventions may not be feasible. Given the importance placed on poverty in the formative research, social and livelihoods interventions may be best placed to address the core needs of both people with psychosis and their caregivers. Such interventions may be best delivered in collaboration with, rather than within, health systems [37]. Finally, our previous research showed that in rural Ethiopia, in common with other LMIC, family members have a prominent role in their relative’s care and treatment decisions [64]. As has been demonstrated in other African settings, involving caregivers in psychosocial interventions is likely to increase acceptability and feasibility [65, 66].

We therefore propose that assessment of caregiver needs, including mental health and burden, should be incorporated into psychosocial interventions for people with schizophrenia. Though directly addressing the mental health needs of caregivers may be beyond the scope of a psychosocial intervention targeting people with schizophrenia especially in mental healthcare systems with limited capacity, some form of psychoeducation or self-help tools for caregivers could be considered. The planned trial qualitative evaluation will hopefully aid future initiatives by illuminating which aspects of CBR impacted on burden and where the gaps are: for example, whether the light touch support provided directly to caregivers was helpful, and why the family support groups did not gain traction. Workers delivering psychosocial interventions such as CBR should be mindful of the greater likelihood of burden and depression in caregivers of people with high levels of disability and should offer additional support and/or referrals as needed. Workers should also be aware of the greater likelihood of high burden and depression amongst children and spouses of people with schizophrenia. CBR workers should be aware of the time burden of CBR participation and fit sessions around participants and caregivers’ schedules, particularly if it is not possible for them to do the work simultaneously. The forthcoming qualitative evaluation will shed light on whether caregivers’ participation in earlier phases of CBR actually decreased their ability to work and whether the benefits of CBR were perceived as an acceptable pay off.

The importance of reducing stigma in mental health was recently highlighted by the Lancet commission on ending stigma and discrimination in mental health [67]. This commission also highlighted the knowledge gap on strategies to reduce stigma in LMIC. Social contact anti-stigma approaches, co-produced and delivered with people with lived experience [68], could be a candidate area for development in future iterations of CBR for schizophrenia. Our findings suggest incorporating caregivers into stigma reduction efforts could be beneficial.

## Conclusions

We previously confirmed the positive effect of CBR on two subdomains of caregiver burden, but the present study did not show such an effect on depression, stigma, employment or other aspects of burden. Caregivers of people with high levels of disability have the highest levels of need. As a result of CBR, caregivers may also reduce their own work due to caring, possibly due to the time commitment of participation. Future research should explore how caregivers’ needs can be best addressed within psychosocial interventions targeting people with schizophrenia, by for instance integrating stigma reduction and social and livelihoods interventions. Investment in sustainable mental healthcare systems in LMIC is key to ensure the wellbeing of people with schizophrenia and their caregivers.

## Electronic supplementary material

Below is the link to the electronic supplementary material.

Supplementary Material 1

## Data Availability

The datasets used and/or analysed during the current study are available from the corresponding author on reasonable request.

## Abbreviations

95%-CI: 95% confidence interval
aOR: Adjusted Odds Ratio
CBR: Community Based Rehabilitation
COPSI: COmmunity-based intervention for People with Schizophrenia and their caregivers in India
FIS: Family Interview Schedule
GEE: Generalised Estimating Equation
ICC: Intra-Cluster Correlation
IEQ: Involvement Evaluation Questionnaire
IQR: Inter Quartile Range
LMIC: Low and Middle Income Countries
LSHTM: London School of Hygiene and Tropical Medicine
MD: Mean Difference
mhGAP-IG: WHO mental health Gap Action Programme-Intervention Guide
N: Number
N/A: Not applicable
OR: Odds Ratio
PHQ-9: 9 item Patient Health Questionnaire
PRIME: PRogramme for Improving Mental healthcarE
RISE: Community-based Rehabilitation Intervention for people with Schizophrenia in Ethiopia
SD: Standard Deviation
SMD: Standardized Mean Difference
SMI: Severe Mental Illness
WHO: World Health Organisation
WHODAS: World Health Organisation Disability Assessment Schedule
β: Beta coefficient for linear regression models
