# Supplementary material for: Effects of community-based rehabilitation on caregivers of people with schizophrenia in Ethiopia in the RISE trial
Source: BMC Psychiatry. 2025 Mar 11;25:231. doi: 10.1186/s12888-025-06651-4 (PMC11899427; doi:10.1186/s12888-025-06651-4)
Supplement: Supplementary file 1 — Supplementary Material 1 [file 12888_2025_6651_MOESM1_ESM.docx]

# Appendix

**A1: Caregiver outcomes and instruments**

| **Outcome** | **Instrument** | **Timepoint** | **Analysis** | **Psychometric properties** |
| --- | --- | --- | --- | --- |
| Depression | 9 item Patient Health Questionnaire (PHQ) | 6 months | Both binary (PHQ>4) and continuous (total PHQ-9 score) | - Validated in Ethiopia (1)  - Cronbach’s α = 0.81-0.84*(2, 3) - ICC=0.92(2)** |
| Stigma | WHO Family interview Schedule (FIS) Stigma section | 6 and 12 months | Continuous (total FIS Stigma score) | - Previously used in Ethiopia  - For Ethiopian caregivers of children with developmental disabilities Cronbach's α =0.92(4, 5)* |
| Burden | WHO Family interview Schedule (FIS) Impact section | 6 and 12 months | Continuous (total FIS Impact score) | - Previously used in Ethiopia(6) |
|  | Involvement Evaluation Questionnaire (IEQ) | 6 and 12 months | Continuous (total IEQ score and scores on the subscales) | - Cronbach’s α = 0.89 in Ethiopia*(7) - ICC>0.7 in a European population (8, 9) ** |
| Reduced work due to caregiving | Single question | 6 months | Binary (those who have and have not reduced their work due to caring) | N/A |
| Employment | Single question | 6 and 12 months | Binary (those who do and do not earn money) | N/A |

*Cronbach’s α is a measure of internal consistency of a scale. **ICC is measure of test-retest reliability.

## A2: Overview of used variables

| **Variable (A-Z)** | **Variable STATA code name** | **Coding** | **Explanation** |
| --- | --- | --- | --- |
| Caregiver age | cgage_3cat | 1= <25 2= 25-35 3= <35 | The caregivers age in years |
| Caregiver different at endline evaluation | diffcaregiverend | 0=Same endline and baseline caregiver 1=Different endline and baseline caregiver | This variable indicated whether the caregiver that completed the endline interview was a different person from the one at baseline |
| Caregiver different at midline evaluation | diffcaregivermid | 0=Same midline and baseline caregiver 1=Different midline and baseline caregiver | This variable indicated whether the caregiver that completed the midline interview was a different person from the one at baseline |
| Caregiver education | cgedu_3cat_eth | 1= illiterate  2= can read and write  3= formal education | This variable categorizes the caregivers education by literacy |
| Caregiver education in years | cgedu_3catsec | 0= 0 years of education  1= 1-8 years of education  2= 9-15 years of education | This variables indicates how many years of education the caregiver has had |
| Caregiver employment | cgempnow_2cat | 0=Employed  1=Not employed | This variable indicates whether the caregiver was employed |
| Caregiver endline follow-up | cgbetween10wks | 0=Within 10 week window  1=Outside 10 week window | This variable indicates whether the endline interview with the caregiver took place within the pre-specified 10 week time window |
| Caregiver family income per month | cg_famincome | 0=<500 birr 1=501-999 birr  2=1000+ birr | This indicates the monthly income of the caregivers family in birr (Ethiopia’s currency) |
| Caregiver FIS impact score | fisimpactpastsum | Continuous | This variable is the summed score on the Impact subscale of the Family Interview Schedule, which is a measure of the caregivers burden. |
| Caregiver FIS stigma score | fisstigmatotalM | Continuous | This variable is the summed score on the Stigma subscale of the Family Interview Schedule, which is a measure of the level of stigma experiences by the caregivers. |
| Caregiver IEQ supervision | ieqsup | Continuous | This variable is the total score on the supervision subscale of the Involvement Evaluation Questionnaire (IEQ). |
| Caregiver IEQ tension | ieqtension | Continuous | This variable is the total score on the tension subscale of the Involvement Evaluation Questionnaire (IEQ). |
| Caregiver IEQ tension - binary | ieqtensionbin | 0= <10  1= >10 | This variable is a binary recode of the IEQ tension score. |
| Caregiver IEQ total | ieqtotalM | Continuous | This variable is the total score on the Involvement Evaluation Questionnaire (IEQ), which is a measure of caregiving burden. |
| Caregiver IEQ urge | iequrge | Continuous | This variable is the total score on the urging subscale of the Involvement Evaluation Questionnaire (IEQ). |
| Caregiver IEQ worrying | ieqworrM | Continuous | This variable is the total score on the worrying subscale of the Involvement Evaluation Questionnaire (IEQ). |
| Caregiver IEQ worrying-binary | ieqworrMbin | 0= <10  1= >10 | This variable is a binary recode of the IEQ worrying score. |
| Caregiver midline follow-up | cgbetween10wksmid | 0 =Within 10 week window 1=Outside 10 week window | This variable indicates whether the midline interview with the caregiver took place within the pre-specified 10 week time window |
| Caregiver occupation | cgocc | 0=unemployed, home worker, or pensioner  1=all other occupations | This variable indicates whether the caregiver worked from home or was mostly at home or whether they had an occupation outside the home. It is a recode of a more detailed variable indicating the caregivers occupation. |
| Caregiver PHQ depression | cgphqpos | 0=PHQ score <4  1=PHQ score >4 | This variable indicates whether the caregiver had a PHQ-9 score above 4, which is an indication of depression. |
| Caregiver reduced work due to caring | reducework | 0= Caregiver did not stop or reduce work due to caring in the past month  1= Caregiver stopped or reduced work due to caring in the past month | This variable indicates whether the caregiver had reduced their work to care for the participant or due to the participants problems. It is a question to measure the caregiving burden. |
| Caregiver relation to pt | child | 0= other  1=child | This variables indicates whether the caregiver was the participants child. |
| Caregiver relation to pt | otherrel | 0= parent, child, spouse, or sibling  1=other | This variables indicates whether the caregiver had another relationship than parent, child, spouse, or sibling to the participant. |
| Caregiver relation to pt | parent | 0= other  1=parent | This variables indicates whether the caregiver was the participants parent. |
| Caregiver relation to pt | relation | 1=parent  2= sibling  3= Child  4= spouse  5= Other | This variable indicates the relationship to the participant. |
| Caregiver relation to pt | sibling | 0= other  1=sibling | This variables indicates whether the caregiver was the participants sibling. |
| Caregiver relation to pt | spouse | 0= other  1=spouse | This variables indicates whether the caregiver was the participants spouse. |
| Caregiver sex | cgsex | 0=male  1=female | This variable indicates the caregivers sex. In the hierarchical analysis of factors associated with caregiver outcomes it was used as a proxy measure for gender. |
| Caregiver social support | oslo_2cat | 0= strong or intermediate support  1= poor support | This variable indicates the level of social support experiences by the patient and the caregiver. It is measured by the Oslo-3 Social Support Scale. |
| Caregiver total PHQ | cgtotalphq | Continuous | This variable indicates the caregivers total score on the PHQ-9 score, which is an instrument that assesses depression. |
| Caregivers marital status | cgmarit_3cat | 1=Single 2=Has a partner  3=Divorced/widowed | This variable indicates the caregivers marital status. |
| Caregivers weekly time spent | cg_timespent | 0= <32 hours per week 1=>32 hours per week | This variable indicates how much time the caregiver spent per week with the participant on average. |
| Health care centre | hcid | Integer | The identifying number of the healthcare centre the participant went to. |
| Kebele | kebid | Integer | The identifying number of the Kebele the participant lived in. Kebeles are administrative sub-districts in Ethiopia and were the unit of randomisation in this trial. |
| Participant age | age_3cat | 1= <25 2= 25-35 3= <35 | The participants age in years. |
| Participant alcohol use | audpos | 0=no  1=yes | This variable indicates whether the participant screened positive for alcohol use disorder on the Alcohol Use Disorders Identification Test (AUDIT). A score of >7 is seen as screening positive. |
| Participant BPRSE disorganization | bprse_disorganization | 0= absent to mild  1=moderate to severe | This variable indicates whether on the BPRSE (Brief Psychiatric Rating Scale Expanded Version) there confused thought processes. The BPRSE is a clinician rated scale to assess symptom change in psychiatric patients. This variable was used in the project as a variable to indicate the presence of disorganized symptoms. |
| Participant disease course prior to study | lcs_coursebin | 0=Never psychotic in period  1=Episodic or continuous | This variable indicates whether the participant was psychotic in the 6 months prior to the baseline interview. |
| Participant employment status | empnow_2cat | 0=Employed  1=Not employed | This variable indicates whether the participant was employed. |
| Participant illness duration | durationbin | 0 =< 4 years 1=4+ years | This variable indicates for how long the participant had had schizophrenia prior to the baseline interview |
| Participant number of suicide attempts | suinumberatt | 0= no suicide attempts 1= one or more suicide attempts | This variable indicates the number of suicide attempts the participant had undertaken. |
| Participant sex | sex | 0=male  1=female | This variable indicates the participant’s sex. |
| Participant symptom severity | cgi_severity | 0= not ill to mildly ill  1=moderately ill  2=markedly ill to the most severely ill | This variable indicates the participants symptom severity on the Clinical Global Impressions Scale (CGI) which is a clinician rates scale which provides a global rating of illness severity for psychiatric patients. |
| Participant WHODAS | cgwhodas36_totalcomplex | Continuous | This variable indicates the total score of the participant on the WHO Disability Assessment Schedule (WHODAS), which is a measurement of the level of disability. This was the main trial outcome. The caregivers filled out the WHODAS about the participant. |
| Participant WHODAS binary | cgwhodas36_totalcomplexbin | 0= <40 1=>40 | This variable is a binary recode of the WHODAS score. |
| Place of residence | residence | 0=Urban 1=Rural | This variable indicates whether the participant lived in an urban or rural area. |
| Socioeconomic status | sesbin | 0 =0 to 3 on poverty scale  1= 4 or more on poverty scale | This variable indicates the participants socioeconomic status. Higher poverty scores mean a lower socioeconomic status. |
| Travel time to facility | timetofacility_3cat | 1= <=60 minutes  2= >60 & <=120 minutes  3= >120 minutes | This variable indicates the travel time between the place where the participant lived and the health facility. |
| Treatment arm | arm | 0= FBC  1= FBC + CBR | This variable indicates whether the participants kebele had been allocated to the CBR intervention or to the control arm. |

## A3: Supplementary methods for validity checks and sensitivity analysis

The validity of the adjusted models was checked using the quadrature approximation for the random-effects logistic regression and by plotting the residuals of the models with a continuous outcome using histograms, plots of the quantiles of residuals against the quantiles of the normal distribution, and scatterplots. As residual plots are not possible with random-effects models, this was done with a linear model where sub-district was added as a covariate and the assumption was made that the residuals would approximate those of the random effects model. For the logistic regression models, a difference between the quadrature points of more than 0.01% was considered as an indication that the model was unreliable and meant the models were done as generalized estimating equation (GEE) models instead.(StataCorp, 2019) For the continuous models where the residuals were not normally distributed, sensitivity analyses were done using a log-transformation on the outcome variable.

**Multiple imputation**

For the multiple imputation, all variables in the final model for the outcome that did not have missing data were used to predict the variables that had missing data. For each of the models, 50 imputations were done. This is in line with the multiple imputation methods used for the main trial paper. The variable for the whether the data collection was done with in the prespecified 10 week window was recoded to have the missing observations (which corresponded with the missing observations for the outcome) coded as the observation being within the window. This was so that the imputed outcome observations could be used in the analysis.

Variables that had a fixed ranged, such as the total FIS stigma score, were truncated in the imputation process, so that no impossible variables would be imputed. Time to facility, which had 3 categories was imputed as an ordered categorical variable. For binary variables that had to be imputed augmented regression was performed in cases where there was perfect prediction. For all imputations, summaries of the first three imputations were inspected to check whether the imputation process worked as anticipated.

For continuous outcomes estimation was set to be allowed when estimation sample varied across imputations. Where this was the care, it is reported in the table with outcomes. For binary outcomes estimation was set to be allowed when errors on some of the imputations occurred, these imputations were then discarded from the analysis. In the case of PHQ score at 6 months, two of the health centres had to be merged as otherwise different observations would have been omitted in the different imputations.

## A4: Factors associated with caregiver outcomes in Ethiopia

| **Factor** | **Outcome*** | **RISE variable used in analysis**** | **Coding**** |
| --- | --- | --- | --- |
| Caregiver gender | - Male = ↓ depression (1)  - Female = ↑burden scores (2),  ↑occupational and financial burden (3), ↑ perceived stigma (4) | Caregiver sex | 0=male  1=female |
| Caregiver level of education | - Secondary level of education= ↓ depression (1)  - No formal education = ↓ burden (2) | Caregiver years of education | 0=0  1=1-8  2=9-15 |
| Age of the caregiver | - Older = ↑ burden scores (2), ↑ stigma scores (5) | Caregiver age in years | 1= <25  2= 25-35  3= >35 |
| Caregiver relation to patient | - Spouse = ↑ burden scores (2), ↑ occupational burden (3), ↑ financial burden (3)  - Children = ↑ family-related burden (3), ↑ burden scores (2)  - Parents= ↑ social burden (3), ↑ financial burden (3)  - Sibling or other relative = ↓ depression (1), ↑ burden scores (2) | Caregiver relationship to patient | 1=parent  2= sibling  3= Child  4= spouse  5= Other |
| The duration of the relationship with the patient | - Longer duration = ↑ perceived stigma (4) | X | X |
| Caregiver marital status | - Separated, divorced or widowed = ↑ financial burden (3), ↑ social burden (3)  - Unmarried caregivers = ↑ perceived stigma (4) | X | X |
| Monthly income | - Higher income = ↓ burden (2), ↓ depression (1) | Income in birr | 0=<500  1= 501-999  2= >1000 |
| Place of residency | - Urban = ↑ perceived stigma (4, 5) | Place of residency | 0=Urban  1=Rural |
| Caregiver attending a place of worship | - Never attending = ↑ depression (1) | X | X |
| Caregiver being a house wife | - Housewife = ↑ depression (1) | Employment of caregiver | 0 =unemployed, home worker, or pensioner 1=all other occupations |
| Perceived stigma | - More stigma = ↑ burden scores (2), ↑ depression (1) | Stigma subscale of the Family Interview Schedule | Summed score as a continuous variable |
| Social support | -High social support = ↓ burden scores (2) (6), ↑ stigma(4), ↓ depression (1) | Oslo-3 Social Support Scale score | 0= not ill to mildly ill 1=moderately ill 2=markedly ill to the most severely ill |
| Time spent caring for patient | - More time spent = ↑ depression (1), ↑ burden scores (2) | time the caregiver spent per week with the participant on average. | 0= <32 hours per week   1. = >32 hours |
| Patients gender | - Female = ↑ social burden (3) | Patient sex | 0=male  1=female |
| Patient education level | - Primary education= ↑ burden scores (2) | Participant years of education | 0=0  1=1-8  2=9-15 |
| Patient employment | - Unemployed = ↑social burden (3), ↑ financial burden (3) | X | X |
| Patient having been admitted | - Admission= ↓ perceived stigma (4) | X | X |
| Illness severity of patient | - Moderately ill = ↑ burden scores (2) | Participant score on the Clinical Global Impressions Scale (CGI) | 0= not ill to mildly ill 1=moderately ill 2=markedly ill to the most severely ill |
| Patient substance use | - Substance abuse = ↑ burden scores (2)  - Alcohol use = ↑ depression (1)  - Khat use = ↑ ability to work (7) | Presence of alcohol use disorder (AUDIT>7) | 0=no  1=yes |
| Negative and positive symptom scores | - Negative and positive = ↑ burden scores (6)  - Negative= ↑ financial burden (3) | Participant score on the Clinical Global Impressions Scale (CGI) | 0= not ill to mildly ill 1=moderately ill 2=markedly ill to the most severely ill |
| Being in remission for over 75% of time | - In remission = ↓ burden (6) | Participant psychosis in the 6 months prior to the baseline interview | 0= never psychotic  1= episodic or continuous |
| Disorganized symptoms | - Disorganization = ↑ social burden, ↑ family burden (3), ↑ occupational and financial burden (3) | BPRSE (Brief Psychiatric Rating Scale Expanded Version), item on confused thought processes. | 0= absent to mild  1= moderate to severe |
| Patient having tried suicide | - Suicide attempt = ↑ depression (1) | Number of suicide attempts by patient | 0= no suicide attempts 1= one or more suicide attempts |

** ↑ indicates a positive association, ↓ indicates a negative association
**X= this variable was not used in the hierarchical analysis, either because it was not in the final framework or because no variable was available for this in the dataset.*

| **Outcome** | **Final model** | **Include outside 10-week window** | **Exclude different caregivers at follow-up** | **Missing confounders left out** | **Multiple imputation** | **HCID 4+5 merged** |
| --- | --- | --- | --- | --- | --- | --- |
| **6 months** | **N=112** | **N=151** | **N=99** | **N=102** | **N=127** | **N=112** |
| **Reduced work due to caring** | 2.40 (1.06-5.45) | 1.66 (1.02-2.71)^b^ | 2.47 (1.11-5.52)^c^ |  | 2.16 (0.89-5.22) |  |
| **Caregiver unemployment** | 2.09 (0.55-7.95) | 1.69 (0.86-3.31) | 1.90 (0.50-7.22) | 2.25 (0.56-8.99) | 2.23 (0.67-7.42) |  |
| **PHQ** | | | | | | |
| >4^a^ | 1.58 (0.52-4.84) | 1.17 (0.52-2.66)^d^ | 1.36 (0.41-4.54)^c^ | 1.38 (0.39-4.91)^e^ | 1.36 (0.45-4.10)^g^ | 1.71 (0.54-5.41) |
| Total score | 0.61 (-0.87-2.09) | 0.34 (-0.80-1.49) | 0.83 (-0.66-2.32) | 0.39 (-0.78-1.56) | 0.48 (-0.82-1.77) |  |
| **FIS** | | | | | | |
| Total stigma | 0.54 (-4.26-5.33) | -0.78 (-3.93-2.36) | 0.52 (-4.37-5.40) | 1.16 (-3.43-5.75)^f^ | -0.14 (-4.40-4.12) |  |
| Total impact | -0.69 (-2.79-1.41) | -0.61 (-2.13-0.92) | -0.54 (-2.85-1.76) | -0.58 (-2.71-1.55)^f^ | -1.14 (-2.96-0.67) |  |
| **IEQ** | | | | | | |
| Urging | -0.76(-4.09-2.58) | -0.24 (-2.69 -2.21) | -0.63 (-4.77-3.52) | -0.40 (-4.18-3.37) | -0.71 (-3.69-2.27) |  |
| Supervision | -1.71 (-4.94-1.53) | -1.61 (-4.03-0.82) | -1.29 (-4.86-2.29) | -1.26 (-4.53-2.02) | -1.49 (-4.30-1.31) |  |
| Worrying | -1.42 (-3.94-1.10) | -0.90 (-3.02-1.21) | -2.00 (-4.75-0.75) | -1.08 (-3.62-1.46) | -1.46 (-3.82-0.89) |  |
| Tension | 1.07 (-1.83-3.97) | 0.54 (-1.98-3.05) | 1.54 (-2.02-5.09) | 1.23 (-1.88-4.33) | 0.95 (-1.57-3.47) |  |
| Total IEQ | -1.67 (-11.65-8.31) | -2.66 (-10.51-5.20) | -0.85 (-11.91-10.22) | -0.16 (-11.03-10.72)^f^ | -2.24 (-11.03-6.56) |  |

## A5: Sensitivity analyses at 6 months *^a^ N=107 ^b^ Convergence was not achieved for this model ^c^ N=95 ^d^ N=146 ^e^ N=98 ^f^ N=101 ^g^ Healthcare center 4 and 5 were merged in this analysis, to prevent different numbers of observations in different imputations.*

## **A6: Sensitivity analyses at 12 months**

| **Outcome** | **Final model** | **Include outside 10-week window** | **Exclude different caregivers at follow-up** | **Missing confounders left out** | **Multiple imputation** |
| --- | --- | --- | --- | --- | --- |
| **12 months** | **N=149** | **N=153** | **N=124** | **N=136** | **N=162** |
| **Caregiver unemployment** | 0.72 (0.35-1.50) | 0.77 (0.38-1.57) | 0.71 (0.27-1.88)^a^ | 0.83 (0.40-1.69) | 0.88 (0.46-1.70) |
| **FIS** | | | | | |
| Total stigma | -2.57 (-6.28-1.13) | -1.59 (-5.43-2.24) | -2.33 (-6.22 -1.56 | -2.26 (-5.94-1.42)^b^ | -2.49 (-6.33-1.36)^c^ |
| Total impact | 0.98 (-0.55-2.52) | 0.75 (-0.76-2.26) | 0.92 (-0.90-2.74) | 0.59 (-0.84-2.02) | 0.88 (-0.54-2.29) |

*^a^ Estimates diverged and estimation could not be made, so WHODAS score was dropped from the model for this estimation. N=121
^b^ N=137*

*^c^ Number of observations different between imputations, N=160-162.*

|  | **N** | **β (95%-CI)** | **p-value** |
| --- | --- | --- | --- |
| **Caregiving coping and capacity** | | | |
| Caregiver age | 138 |  | 0.59 |
| <25 |  | 0 |  |
| 25-35 |  | 3.06 (-6.61-12.73) |  |
| >35 |  | 4.27 (-3.85-12.40) |  |
| Social support | 149 |  | 0.25 |
| Strong to intermediate |  | 0 |  |
| Poor |  | 3.54 (-2.51 – 9.59) |  |
| Caregiver education | 138 |  | 0.01 |
| 0 years |  | 0 |  |
| 1-8 years |  | -6.02 (-12.94-0.90) |  |
| 9-15 years |  | -13.65 (-23.03 - -4.26) |  |
| Income | 148 |  | 0.14 |
| <500 birr |  | 0 |  |
| 501-999 birr |  | 1.66 (-5.57 - 8.90) |  |
| >1000 birr |  | -8.17 (-17.12-0.78) |  |
| **Social norms** | | | |
| Caregiver sex | 138 |  | 0.28 |
| Male |  | 0 |  |
| Female |  | 3.30 (-2.70-9.30) |  |
| Place of residence | 137 |  | 0.68 |
| Urban |  | 0 |  |
| Rural |  | 2.32 (-8.56-13.20) |  |
| Sibling | 149 | 0.02 (-7.03-7.07) | 0.99 |
| Other relative | 149 | -10.47 (-20.52- -0.43) | 0.04 |
| Child | 149 | 1.39 (-5.85-8.64) | 0.71 |
| Spouse | 149 | -0.85 (-8.16 – 6.45) | 0.82 |
| Parent | 149 | 4.75 (-1.78 – 11.28) | 0.15 |

|  | **N** | **β (95%-CI)** | **p-value** |
| --- | --- | --- | --- |
| **Patient factors** | | | |
| WHODAS | 149 | 0.27 (0.15-0.38) | <0.001 |
| Disease course in 6 months before study | 149 |  | 0.34 |
| Never psychotic |  | 0 |  |
| Episodic or continuous |  | 3.76 (-3.92-11.44) |  |
| Disorganization | 147 |  | 0.01 |
| Absent to mild |  | 0 |  |
| Moderate to severe |  | 9.17 (2.30-16.04) |  |
| Disease severity | 149 |  | 0.34 |
| Mild |  | 0 |  |
| Moderate |  | 2.64 (-4.45-9.73) |  |
| Severe |  | 5.57 (-1.85-12.99) |  |
| Education | 138 |  | 0.36 |
| 0 years |  | 0 |  |
| 1-8 years |  | -4.02 (-10.59-2.56) |  |
| 9-15 years |  | -5.75 (-16.57-5.06) |  |
| Alcohol use disorder | 143 |  | 0.75 |
| No |  | 0 |  |
| Yes |  | -1.27 (-9.13-6.58) |  |
| Suicide attempts | 148 |  | 0.82 |
| 0 |  | 0 |  |
| 1 or more |  | -1.39 (-13.20-10.42) |  |
| **FIS stigma** | 149 | 0.52 (0.21-0.84) | 0.001 |
| **Leaving home to work** | 149 |  | 0.05 |
| No |  | 0 |  |
| Yes |  | -5.85 (-11.72-0.02) |  |
| **Time spent with person** | 149 |  | 0.05 |
| <32 hours per week |  | 0 |  |
| >32 hours per week |  | 7.40 (0 -14.80) |  |

## A7: Associations with burden at 12 months

|  | **N** | **β (95%-CI)** |  |
| --- | --- | --- | --- |
| **Caregiver coping and capacity** | | | |
| Caregiver age |  |  | 0.46 |
| <25 |  | 1 |  |
| 25-35 |  | 1.35 (0.45-4.04) |  |
| >35 |  | 1.84 (0.66-5.13) |  |
| Social support | 149 |  | 0.04 |
| Strong to intermediate |  | 1 |  |
| Poor |  | 2.00 (1.02-3.91) |  |
| Caregiver education | 138 |  | 0.12 |
| 0 years |  | 1 |  |
| 1-8 years |  | 0.41 (0.17-0.97) |  |
| 9-15 years |  | 0.46 (0.10-2.04) |  |
| Income | 148 |  | 0.67 |
| <500 birr |  | 1 |  |
| 501-999 birr |  | 0.70 (0.31-1.55) |  |
| >1000 birr |  | 0.98 (0.34-2.84) |  |
| **Social norms** | | | |
| Caregiver sex | 138 |  | 0.02 |
| Male |  | 1 |  |
| Female |  | 2.33 (1.11-4.88) |  |
| Place of residence | 137 |  | 0.04 |
| Urban |  | 1 |  |
| Rural |  | 4.86 (1.08-21.92) |  |
| Sibling | 149 | 0.20 (0.06-0.63) | 0.01 |
| Other relative |  | 0.37 (0.12-1.12) | 0.08 |
| Child |  | 3.75 (1.60-8.78) | 0.002 |
| Spouse |  | 0.74 (0.33-1.67) | 0.47 |
| Parent |  | 2.62 (1.11-6.20) | 0.03 |

|  | **N** | **OR (95%-CI)** | **p-value** |
| --- | --- | --- | --- |
| **Person with schizophrenia factors** | | | |
| WHODAS | 149 | 1.01 (1.00-1.02) | 0.09 |
| Disease course in 6 months before study | 149 |  | 0.69 |
| Never psychotic |  | 1 |  |
| Episodic or continuous |  | 1.17 (0.55-2.48) |  |
| Disorganization | 147 |  | 0.27 |
| Absent to mild |  | 1 |  |
| Moderate to severe |  | 1.61 (0.68-3.78) |  |
| Disease severity | 149 |  | 0.44 |
| Mild |  | 1 |  |
| Moderate |  | 0.64 (0.30-1.37) |  |
| Severe |  | 0.92 (0.42-2.01) |  |
| Education | 149 |  | 0.10 |
| 0 years |  | 1 |  |
| 1-8 years |  | 1.35 (0.65-2.82) |  |
| 9-15 years |  | 0.34 (0.09-1.29) |  |
| Alcohol use disorder | 143 |  | 0.93 |
| No |  | 1 |  |
| Yes |  | 0.96 (0.39-2.38) |  |
| Suicide attempts | 148 |  | 0.67 |
| 0 |  | 1 |  |
| 1 or more |  | 0.76 (0.20-2.80) |  |
| **Burden** | | | |
| Total IEQ | 149 | 1.03 (1.01-1.05) | 0.001 |
| FIS Impact | 149 | 1.15 (1.00-1.32) | 0.05 |
| **FIS Stigma** |  | 1.06 (1.02-1.10) | 0.004 |
| **Time spent with person** | 149 |  | 0.05 |
| <32 hours per week |  | 1 |  |
| >32 hours per week |  | 2.42 (1.00-5.56) |  |
| **Leaving home to work** | 149 |  | 0.15 |
| No |  | 1 |  |
| Yes |  | 0.62 (0.33-1.19) |  |

## A8: Associations with depression at 12 months
